# E3 Ubiquitin Ligase E6AP Negatively Regulates Adipogenesis by Downregulating Proadipogenic Factor C/EBPalpha

**DOI:** 10.1371/journal.pone.0065330

**Published:** 2013-06-07

**Authors:** Pooja Pal, Savita Lochab, Jitendra Kumar Kanaujiya, Isha Kapoor, Sabyasachi Sanyal, Gerhard Behre, Arun Kumar Trivedi

**Affiliations:** 1 LSS008, DTDD Division, CSIR-Central Drug Research Institute, Lucknow, India; 2 Division of Hematology and Oncology, University Hospital of Leipzig, Leipzig, Germany; Northwestern University Feinberg School of Medicine, United States of America

## Abstract

CCAAT/Enhancer Binding Protein Alpha (C/EBPα) is a key transcription factor involved in the adipocyte differentiation. Here for the first time we demonstrate that E6AP, an E3 ubiquitin ligase inhibits adipocyte differentiation in 3T3-L1 cells as revealed by reduced lipid staining with oil red. Knock down of E6AP in mouse 3T3L1 preadipocytes is sufficient to convert them to adipocytes independent of external hormonal induction. C/EBPα protein level is drastically increased in E6AP deficient 3T3L1 preadipocytes while inverse is observed when wild type E6AP is over expressed. We show that transient transfection of wild type E6AP downregulates C/EBPα protein expression in a dose dependent manner while catalytically inactive E6AP-C843A rather stabilizes it. In addition, wild type E6AP inhibits expression of proadipogenic genes while E6AP-C843A enhances them. More importantly, overexpression of E6AP-C843A in mesenchymal progenitor cells promotes accumulation of lipid droplets while there is drastically reduced lipid droplet formation when E6AP is over expressed. Taken together, our finding suggests that E6AP may negatively control adipogenesis by inhibiting C/EBPα expression by targeting it to ubiquitin-proteasome pathway for degradation.

## Introduction

Adipose tissue metabolism is crucial for modulation of metabolic processes and whole body insulin sensitivity homeostasis [1]. The trend of increased obesity and diabetes observed over the past few years has made it essential to get new insights into the understanding of adipocyte biology and adipogenesis per se for novel possibilities of preventive and effective treatment [2]. During adipocyte differentiation, fibroblast-like preadipocytes differentiate into lipid-loaded adipocytes, a highly insulin-sensitive cell type. These preadipocytes differentiate under hormonal induction and there are arrays of transcription factors which execute this process of differentiation [3], [4]. Peroxisome proliferator-activated receptor gamma (PPARγ) [5] and members of the CCAAT/enhancer-binding protein (C/EBP) family of proteins are key factors involved in adipogenesis [6]. C/EBP family of proteins regulate proliferation and differentiation of large number of cell types and were among first to be implicated in adipocyte differentiation [7], [8], [9]. C/EBPα is the founding member of the C/EBP family and is expressed predominantly in post-mitotic cells. Overexpression of C/EBPα in preadipocytes was shown to arrest 3T3-L1 pre-adipocytes in G0/G1 [10], providing the first evidence that C/EBPα can enforce cell-cycle exit. Moreover, Ectopic expression of C/EBPα in mouse fibroblast cell lines has been shown to promote adipogenic differentiation [11]. During adipogenesis C/EBPα expression is induced and it activates genes specifically expressed in differentiated fat cells [12]. C/EBPβ and C/EBPδ are induced early during the adipogenesis, while C/EBPα is induced at a later stage. C/EBPβ and C/EBPδ increase the PPARγ gene expression by binding to its promoter. PPARγ subsequently activates the expression of multiple genes involved in lipogenesis and adipogenesis. PPARγ also activates the C/EBPα gene. Notably, C/EBPα can bind to the PPARγ promoter and enhance the expression of PPARγ, thereby creating a proadipogenic feed-forward loop (13).

E6AP, a 100 kDa cellular protein belongs to a class of functionally related E3-ubiquitin-protein ligases defined by the domain homologous to the carboxy terminus (HECT) domain [13]. E3 ligases are known to ubiquitinate and degrade various transcription factors [14] and play major role in the regulation of cell cycle and various other cellular functions. E6AP has been reported to play role in the ubiquitination of various proteins like p53, p27, PML-RARα which play important role as tumor suppressors and cell proliferation inhibitors [15], [16], [17]. In recent years some of ubiquitin ligases have been implicated in the modulation of adipogenesis. Fbwx7 degrades C/EBPα and negatively regulates adipogenesis [18] while Skp2 has been shown to mediate p27 degradation during S/G2 phase progression of adipocyte hyperplasia [19]. In a recent report, using mass spectrometry based proteomics approach we identified E6AP as a target of tamoxifen. In addition, previously, we and others have also shown that C/EBPα can be ubiquitinated and degraded via proteasomal pathway [20]. However, to our knowledge, except Fbwx7 no other E3 ligase for C/EBPα ubiquitination is known.

We hypothesized that E6AP may target C/EBPα for degradation. C/EBPα is a transcription factor involved in the regulation of granulopoiesis, in fact, in a recent study we have shown that E6AP promotes C/EBPα ubiquitination leading to its proteasome mediated degradation and thereby inhibits granulopoiesis in blood cells (data not shown). As C/EBPα plays an important role in adipocyte differentiation we further explored its role during adipogenesis in the presence and absence of E6AP. In the present study, we show that E6AP targets C/EBPα for ubiquitin mediated degradation in 3T3L1 cells and thus inhibits adipogenesis. In contrary, E6AP knock down in 3T3L1 cells by siE6AP and over expression of catalytically inactive E6AP-C843A induces adipogenesis. Taken together, our data suggests that E6AP may negatively regulate adipogenesis by targeting C/EBPα for degradation.

## Materials and Methods

### Ethics Statement

Study involving animal experiments was carried out in strict accordance with CPCSEA/IAEC (Registration No.: 34/1999 dated 11-3-99) guide lines. The study involving use of C57Bl/6J mice was approved by the Institutional Animal Ethics Committee at Central Drug Research Institute. Approval reference no. for this study was IAEC/2012/47, dated 16.05.2012.

### Cell Culture and Expression Plasmids

Mouse preadipocyte cell line 3T3L1 was cultured in DMEM supplemented with 10% FBS and antibiotics. Mouse mesenchymal progenitor cells were isolated from bone marrow of C57Bl/6J mice and were cultured in DMEM supplemented with 10% FBS and antibiotics. Cells were incubated in 5% CO2 humidified chamber.

### Plasmids and siRNA

Expression plasmids for pcDNA3.1-E6AP [15] and pCAG-HA-E6AP-C843A [21] were kind gifts from Nihar Jana [15] and Ikuo Shoji [21] respectively. The siE6AP and scrambled siRNA were purchased from Dharmacon. E6AP-C843A is a catalytically inactive form of E6AP where active site cysteine residue is substituted with alanine (C843A). This cysteine residue present in the catalytic domain transfers ubiquitin directly to the substrate via ubiquitin-enzyme cascade leading to their degradation.

### Western Blotting

Cells were harvested after indicated time points using RIPA buffer (1% NP40, 0.5% Sodium deoxycholate, 0.1% SDS, 0.15M NaCl, 5 mM EDTA and 50 mM Tris pH8.0) and equal amount of proteins were separated on 10% SDS-PAGE as previously described [20], [22]. Subsequently proteins were transferred and immunoblotted using primary antibodies against C/EBPα, β actin (Santacruz biotechnology) and E6AP (Sigma).

### Oil Red O Staining

Fully confluent 3T3L1 cells were stimulated with 0.5 mM methylisobutylxanthine, 1 uM dexamethasone and 10 ug/ml insulin (MDI) in DMEM supplemented with 10% FBS to induce differentiation. After 48 h, culture medium was replaced with DMEM supplemented with 10% FBS and 10 ug/ml insulin for additional 48 h. Cells were then fed every other day with DMEM containing 10% FBS. After cells were fully differentiated, Oil red O staining was performed by fixing the cells in 10% formalin. Oil red O stain was prepared in water and was filtered with 0.22 u filter. After staining cells with Oil red O for 10 minutes, cells were washed thrice with water and were air dried. Cells were photographed under light microscope (Leica).

### *In vivo* Ubiquitination Assay

It was performed as previously described [23]. Briefly, 3T3-L1 preadipocytes pre-treated with MDI for 48 h were transiently transfected with expression plasmids for E6AP, E6AP-C843A and His-ubiquitin as indicated. Post 48 h transfection, whole cell extracts (WCEs) were prepared and endogenous C/EBPα was co-immunoprecipitated using 3 ug of C/EBPα antibody and protein G Agarose beads (Millipore). The co-immunoprecipitates were then resolved on 8% SDS-PAGE and immunoblotted with anti-His antibody. Same blot was stripped and reprobed with anti- C/EBPα antibody to assess and confirm the expression and immunoprecipitation of endogenous C/EBPα.

### Quantitative PCR (Real Time) Analysis

3T3L1 cells were transfected with siE6AP and siRNA control, 48 h post transfection, cells were treated with MDI until ten days and subsequently RNA was isolated using Trizol reagent as previously described [24]. Further, RNA was retro-transcribed in to cDNA and subsequently used for quantitative PCR analysis on Roche Light Cycler 480 using SYBR green master mix from Applied Biosystems. Statistical analysis was performed using ΔΔ CT method. Primers used for Real time PCR are listed in table 1.

**Table 1:** List of Primers.

S.No.	Primer	Sequence
1	C/EBP alpha Forward Primer	5′-AGCAACGAGTACCGGGTA-3′
2	C/EBP alpha Reverse Primer	5′-TGTTTGGCTTTATCTCGG-3′
3	PPAR-gamma Forward Primer	5′-CAAGAATACCAAAGTGCGATCAA-3′
4	PPAR-gamma Reverse Primer	5′-GAGCTGGGTCTTTTCAGAATAATAAG-3′
5	SREBP1C Forward Primer	5′-GATCAAAGAGGAGCCAGTGC-3′
6	SREBP1C Reverse Primer	5′-TAGATGGTGGCTGCTGAGTG-3′
7	Adipsin Forward Primer	5′-TGCACAGCTCCGTGTACTTC-3′
8	Adipsin Reverse Primer	5′-CACCTGCACAGAGTCGTCAT-3′
9	Leptin Forward Primer	5′-AGCAGTGCCTATCCAGAAAGT-3′
10	Leptin Reverse Primer	5′-TTCTCCAGGTCATTGGCTAT-3′

### Isolation of Mesenchymal Progenitor Cells

Mesenchymal stem cells were isolated using established protocol [25]. This study was carried out in strict accordance with CPCSEA/IAEC (Registration No.: 34/1999 dated 11-3-99) guide lines. The study involving use of C57Bl/6J mice was approved by the Institutional Animal Ethics Committee at Central Drug Research Institute. Approval reference no. for this study was IAEC/2012/47, dated 16.05.2012. Briefly, bone marrow aspirates were flushed out from tibia and femur, mixed with MACS buffer and loaded on histopaque-1077 (Sigma) gradient to harvest cells from the interphase. Isolated cells were washed with MACS buffer (1X PBS, 2 mM EDTA and 2% FBS) and seeded for expansion in complete isolation medium that constituted of Dulbecco’s modified Eagle’s medium (DMEM) supplemented with 10% FBS and antibiotics [100 units/ml penicillin and 100 µg/ml streptomycin (Invitrogen)] at 37°C in 5% CO_2_ humidified chamber. 48 h post culture, non-adherent cells were carefully removed and adherent mesenchymal stem cells were washed with DMEM and placed in fresh medium. Cells were grown till they reached 60–70% confluence followed by trypsinization for sub-culturing to obtain a purified population of mesenchymal progenitor cells. Medium was changed two times a week.

## Results

### E6AP Negatively Regulates Adipogenesis

**Figure 1:**
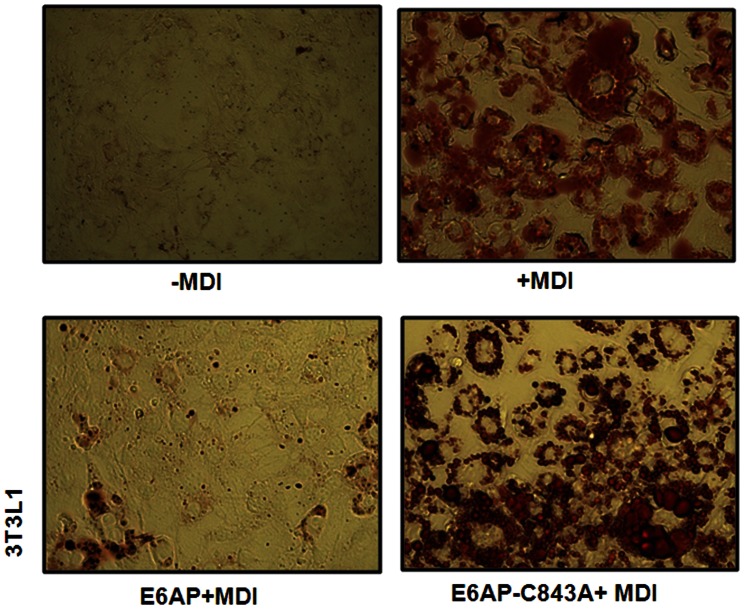
E6AP negatively regulates Adipogenesis. 3T3L1 cells were transfected with E6AP (2.0 µg) and E6AP-C843A (2.0 µg). Post 48 h of transfection, cells were treated with MDI for next 8 days followed by Oil red O staining.

In recent years some of E3 ligases have been shown to be involved in the regulation of adipogenesis [15], [16], [19]. In the present study we have investigated if E6AP, an E3 ubiquitin ligase also modulates adipogenesis. To address this, we transiently transfected 3T3-L1 preadipocytes with wild type E6AP and a dominant negative mutant E6AP-C843A [21]. Over expression of E6AP potentially inhibited adipogenesis even in the presence of adipogenic factors (MDI: 0.5 mM Methylisobutylxanthine, 1 uM Dexamethasone and 10 ug/ml Insulin), whereas no inhibition was seen with E6AP-C843A; Intensity of staining correlated with extent of adipogenesis induction (Fig. 1).

This result suggests that E6AP has inhibitory effect on the differentiation of 3T3-L1 cells even in the presence of hormonal induction. To further explore the role of E6AP on the adipogenesis we studied its effect on an important proadipogenic factor C/EBPα as in a recent study we have shown that E6AP targets C/EBPα for ubiquitin mediated degradation [26].

### E6AP Downregulates Proadipogenic Factor C/EBPα

C/EBPα is a key transcription factor involved in the regulation of adipogenesis. We and others have previously shown that C/EBPα can be ubiquitinated and degraded via proteasome mediated pathway [20], [27]. Since our data suggest that E6AP, an E3 ubiquitin ligase negatively regulates adipogenesis, we sought to assess if E6AP modulates stability of C/EBPα protein.

**Figure 2:**
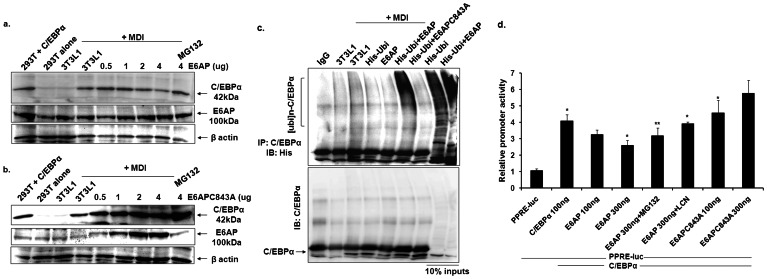
E6AP downregulates expression of proadipogenic factor C/EBPα. (**a,b**) 3T3-L1 preadipocytes were transfected with increasing amounts of E6AP (0.5 µg–2.0 µg) and E6AP-C843A (0.5 µg–2.0 µg). Post 48 h transfection, WCEs were prepared and resolved on 10% SDS-PAGE followed by immunoblotting with C/EBPα, E6AP and β actin antibodies; lysates of 293T alone and transfected with C/EBPα were used as positive and negative control, note that there is no endogenous expression of C/EBPα in 293T. **E6AP promotes C/EBPα degradation through ubiquitin-proteasome pathway:** (**c**) 3T3-L1 preadipocytes pre-treated with MDI for 48 h were transiently transfected with expression plasmids for E6AP, E6AP-C843A and His-ubiquitin. Post 48 h Transfection, WCEs were prepared and C/EBPα was co-immunoprecipitated. IgG was used as a control. Co-immunoprecipitates were resolved on 8% SDS-PAGE and blots were probed with anti-His and anti-C/EBPα antibody respectively. **E6AP inhibits C/EBPα transactivation potential to activate PPRE-Luc:** (**d**) E6AP mediated downregulation of C/EBPα curtails its transactivation potential: 3T3-L1 preadipocytess were transiently transfected with pPPRE-luc reporter and expression plasmids for C/EBPα, E6AP and E6AP-C843A. 24 h post transfection, luciferase activity was measured. MG132 and lactacytin (LCN) treatment was given 3 h prior to cell harvesting for luciferase activity measurement. Data are representative of three independent experiments. Results are given as standard error of mean (± s.e.m.); *p<0.05; **p<0.001, ***<0.0001.

We sought to assess C/EBPα protein expression in 3T3-L1 preadipocytes by transiently transfecting increasing amounts of E6AP or E6AP-C843A (0.5 ug–4.0 ug). Post 48 h transfection, cell lysates were prepared and resolved on 10% SDS-PAGE (Note that cells were induced with MDI for just 2 days for inducing expression of C/EBPα prior to transfection). Immunoblot with C/EBPα antibody shows that E6AP over expression led to substantial decrease in endogenous C/EBPα protein expression while it was rather stabilized in E6AP-C843A transfected condition (fig. 2a, b). Notably, C/EBPα protein expression was restored in MG132 treated cells (in 4.0 ug E6AP transfected 3T3-L1 cells) which indicates that C/EBPα protein stability seems to be regulated via proteasomal pathway.

To further consolidate the notion that E6AP destabilizes C/EBPα protein expression by promoting its ubiquitination and subsequent protasome mediated degradation, we performed *in vivo* ubiquitination assay. For this, 3T3L1 preadipocytes prior induced with MDI for 48 h were co-transfected with expression plasmids for E6AP, E6AP-C843A and His-Ubiquitin. Post 48 h transfection, WCEs were prepared and C/EBPα was co-immunoprecipitated with anti-C/EBPα antibody. The co-precipitates were resolved on 8% SDS-PAGE and immunoblotted with anti-His antibody. Heavy C/EBPα ubiquitination as a ladder was observed in E6AP and His-Ubiquitin co-transfected cells while almost no ubiquitination was seen in cells co-transfected with E6AP-C843A and His-Ubiquitin suggesting E6AP indeed promotes ubiquitination of C/EBPα leading to its degradation via proteasome pathway. Same blot was stripped and probed with C/EBPα antibody to confirm the presence of C/EBPα in the immunoprecipitates (fig. 2c).

### E6AP Mediated Downregulation of C/EBPα Negatively Affects its Transactivation Potential

Since E6AP downregulates C/EBPα protein expression by promoting its degradation, we asked if this E6AP mediated downregulation of C/EBPα has any affect on C/EBPα transactivation potential. To answer this, we performed luciferase reporter assay on a minimal PPRE-Luc promoter containing PPRE response elements. Indicated amounts of reporter vector and expression plasmids for C/EBPα, E6AP and E6AP-C843A were transfected in 293T cells. Post 24 h transfection, luciferase activity was measured which showed that co-transfection of E6AP with C/EBPα substantially inhibited C/EBPα transactivation capacity in a dose dependent manner (fig. 2d). Further, MG132 and Lactacystin (LCN) treatment efficiently restored C/EBPα transactivation potential even in the presence of E6AP. Additionally, co-transfection of E6AP-C843A with C/EBPα did not inhibit transactivation potential of C/EBPα. This data indicates that catalytically active E6AP negatively modulates C/EBPα protein stability thereby hampers its functions.

### siE6AP Promotes Adipogenesis

**Figure 3:**
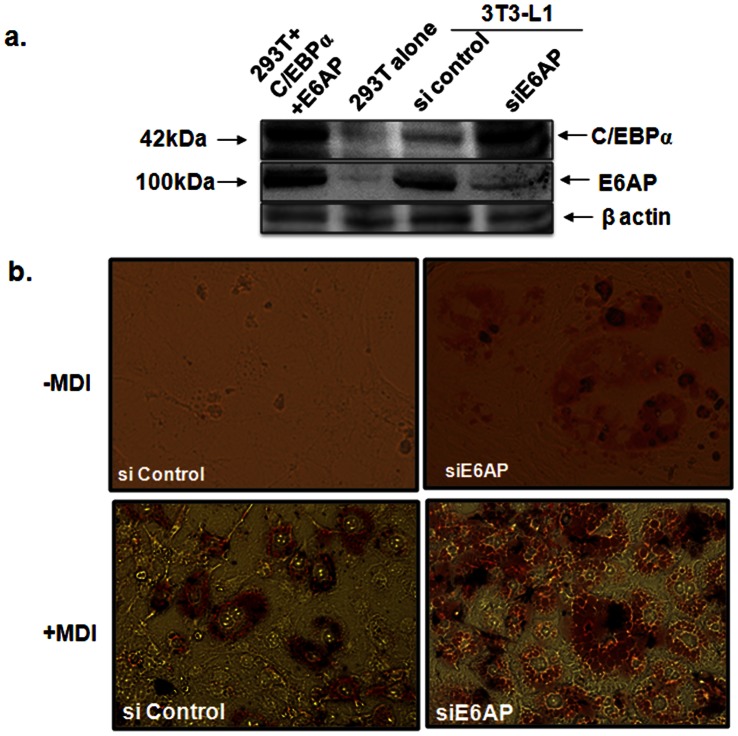
E6AP knock down by siE6AP promotes adipogenesis. (**a**) 3T3L1 preadipocytes pre-treated with MDI were transfected with siE6AP or scrambled siRNA, 48 h post transfection, cells were harvested, resolved on 10% SDS-PAGE and probed C/EBPα, E6AP and β-actin antibody (lysates of 293T transfected with C/EBPα were used as control) (**b**) 3T3L1 preadipocytes were transfected with siE6AP or scrambled siRNA. Cells were grown in the presence or absence of MDI for 10 days followed by Oil red O staining.

Since overexpression of E6AP led to decrease in C/EBPα protein expression while catalytically inactive E6AP stabilized it, we sought to assess if inhibition of E6AP via siRNA against E6AP stabilizes C/EBPα protein expression. 3T3-L1 preadipocytes pre-treated with MDI for 48h were transiently transfected with control siRNA and siE6AP; 48 h post transfections WCEs were prepared and resolved on 10% SDS-PAGE. Immunoblot with C/EBPα and E6AP antibody showed that siE6AP potentially reduced E6AP expression with concomitant increase in C/EBPα expression (fig. 3a). This makes a very interesting observation and confirms our hypothesis that E6AP acts as negative regulator of adipogenesis probably by targeting C/EBPα.

In order to further corroborate the anti-adipogenic role of E6AP, we used siRNA against E6AP. 3T3-L1 cells were transfected either with control siRNA (Scrambled) or siE6AP and were further grown for 10 days in DMEM with or without MDI. Post 10 day transfection; cells were stained with Oil red O staining. As shown in fig. 3b; as compared to control siRNA, siE6AP transfected preadipocytes differentiated into adipocytes showing accumulation of large number of intracellular lipid droplets in response to MDI treatment. Notably, siE6AP transfection without MDI treatment also led to substantial intracellular lipid droplet accumulation which suggests that E6AP inhibition per se may induce adipogenesis.

### E6AP Knockdown Mediated Induction of Adipogenesis is Marked by Increase in Expression of Proadipogenic Factors

**Figure 4:**
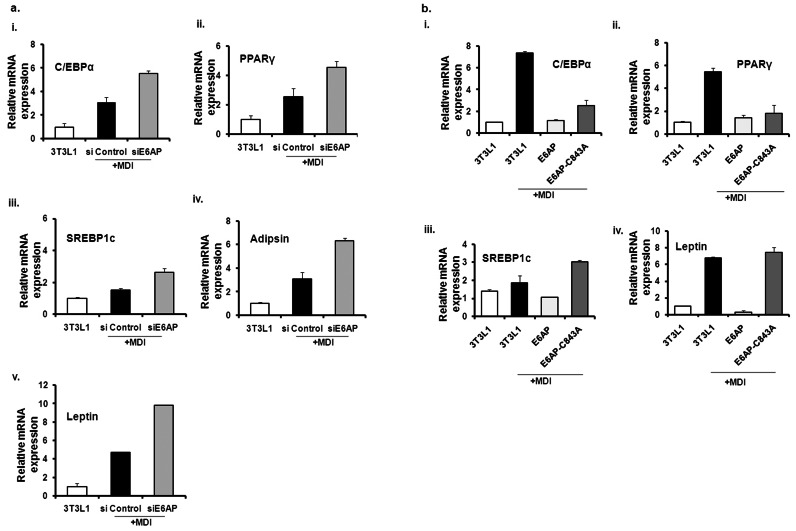
siE6AP mediated induction of adipogenesis is marked by increase in expression of proadipogenic factors. (**a**) 3T3L1 cells were transfected with siE6AP or scrambled siRNA. Post 48 h transfection, cells were treated with MDI for until ten days; subsequently mRNA expression levels of indicated adipogenic genes was determined with qRT-PCR. **Over expression of wild type E6AP down regulates while catalytically inactive E6AP-C843A up regulates proadipogneic factors:** (**b**) 3T3L1 cells were transfected with E6AP and E6AP-C843A. After transfection cells were treated with MDI for 10 days and mRNA expression level of various adipogenic genes was determined with real time PCR. The primers used for C/EBPα, PPARγ, Leptin, SREBP1c and Adipsin listed in table 1.

siE6AP transfection in 3T3-L1 cells enhances lipid droplet formation both in the presence and absence of MDI treatment. This suggests E6AP to be a negative switch in the process of adipogenesis. We therefore asked if this enhanced lipid droplet formation (adipogenesis) is also marked with increase in the expression of adipogenic markers (C/EBPα, PPARγ, SREBP1c and Leptin). PPARγ and C/EBPα are known to function co-operatively to transactivate adipocyte genes and thereby bring about adipocyte differentiation [28]. Leptin is secreted by adipocytes and it may function in regulating body fat mass. Leptin levels are elevated in human obesity and animal models of obesity [29]. Role of SREBP1c in adipogenesis is suggested by its mRNA level increase during adipocyte differentiation where it further transactivates other lipogenic genes [8] [30]. We performed real time PCR for indicated adipogenic genes from mRNA isolated from different conditions as mentioned in fig. 4a. Expression of adipogenic markers (C/EBPα, PPARγ, SREBP1c, Leptin and Adipsin) in siE6AP transfected cells treated with MDI compared to control correlated well with enhanced intracellular lipid droplet formation. This increase in the expression of proadipogenic factors in the presence siE6AP as compared to control siRNA treated with MDI indicates E6AP inhibition is required to drive adipogenesis.

### Over Expression of Wild Type E6AP Down Regulates while Catalytically Inactive E6AP-C843A Up Regulates Proadipogenic Factors

E6AP inhibits C/EBPα protein expression leading to its functional inactivation (fig. 3 and 4); moreover, proportion of lipid droplet formation varies in E6AP and E6AP-C843A transfected 3T3-L1 cells treated with MDI. In addition, we showed that siE6AP transfection enhanced mRNA expression levels of proadipogenic factors; therefore, we further assessed the expression of these adipogenic markers (C/EBPα, PPARγ, SREBP1c and Leptin) in 3T3-L1 cells transfected with E6AP and E6AP-C843A. As shown in fig. 4b mRNA expression levels of these proadipogenic factors correlated well with proportion of lipid droplet formation in E6AP and E6AP-C843A transfected cells treated with MDI (fig. 1). Taken together these data endorse our finding that E6AP negatively regulates adipogenesis.

### E6AP Over Expression Inhibits while E6AP-C843A Promotes Adipocyte Differentiation of MDI Treated Mesenchymal Stem Cells Isolated from Murine Bone Marrow

**Figure 5:**
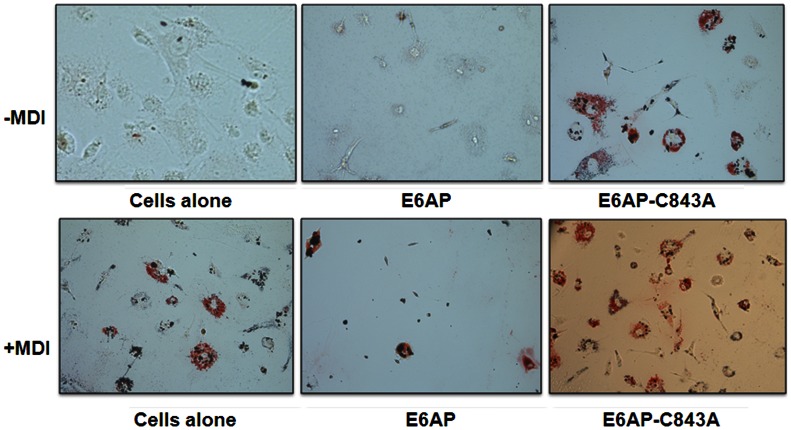
E6AP over expression inhibits while E6AP-C843A promotes adipocyte differentiation of MDI treated mesenchymal stem cells isolated from murine bone marrow. Mesenchymal progenitor cells were transfected with E6AP (2.0 µg) and E6AP-C843A (2.0 µg). Post 48 h of transfection, cells were grown in presence or absence of MDII for next 8 days followed by Oil red O staining.

In order to further corroborate our finding in a more physiological setting, we isolated mouse mesenchymal progenitor cells from bone marrow of C57Bl/6J mice. These mesenchymal progenitor cells can differentiate in adipogenic or osteogenic lineage depending upon the kind of hormonal inductions. Therefore, we separately over expressed both E6AP and E6AP-C843A in these progenitors cells and cultured in DMEM in the presence or absence of MDII (methylisobutylxanthine, dexamethasone, insulin and indomethacin). Post 10 days of transfection and culture in MDII, Oil red O staining was performed to assess the adipogenic potential of these transfected cells. Intracellular lipid droplet formation was observed in the mesenchymal progenitor cells transfected with E6AP-C843A mutant even in the absence of MDII induction. However, in E6AP transfected cells lipid droplet formation was inhibited even in the presence of MDII (fig. 5). This further consolidates our finding that E6AP negatively regulates adipogenesis apparently by downregulating proadipogenic protein C/EBPα.

## Discussion

C/EBPα is an important transcription factor involved in the regulation of various cellular processes including adipogenesis. E3 Ligases have attracted great interest because of their role in a variety of cellular processes by virtue of their ability to target regulatory proteins for degradation. In recent years, some of E3 ligases have also been implicated in the regulation of adipogenesis. Fbwx7 targets C/EBPα for phosphorylation dependent ubiquitin mediated degradation and thereby negatively regulates adipogenesis [18]. Expression of Fbxw7 is down-regulated early during adipogenesis, leading to the stabilization of C/EBPα, which in turn results in enhanced expression of proadipogenic target genes and enhanced adipogenesis. In addition, Skp2 has been shown to mediate p27 degradation during S/G2 phase progression of adipocyte hyperplasia [19]. HECT (homologous to the E6AP C terminus) ubiquitin ligases, like E6AP possess E3 ligase activity and are involved in diverse functions in eukaryotic cells, largely through their ability to ubiquitinate the target proteins thereby affecting their localization, interactions, and stability. Recently, HECT domain containing E3 ubiquitin Liagse WWP1 was implicated in lipid droplet turnover. WWP1 interacts with and ubiquitinates lipid droplet binding protein Spartin/SPG20. SPG20 in conjunction with TIP47 regulates lipid droplet number as well as size [31].

Here for the first time we show that a HECT domain containing E3 ubiquitin ligase E6AP inhibits adipogenesis by downregulating proadipogenic factor C/EBPα. Since C/EBPα is a master transcription factor that regulates number of genes required for adipogenic differentiation switch, proteins targeting this master regulator may be very crucial in regulating adipogenesis. E6AP seems to be one such crucial protein since it inhibits adipogenesis by downregulating C/EBPα protein expression (fig. 1).

Our data is further substantiated by finding that siE6AP mediated knock down of E6AP is sufficient to induce adipocyte differentiation in 3T3-L1 cells even in the absence of hormonal treatment (fig.2). Considerable knock down of E6AP in preadipocyte 3T3-L1 cells led to accumulation of C/EBPα protein and subsequent increased intracellular lipid droplet formation. This provides clear evidence regarding direct correlation between E6AP and C/EBPα activity because increase in lipid droplet formation (a direct measure of adipogenesis) is in fact induced by C/EBPα. This clearly demonstrates that E6AP is an inhibitory factor for the induction of adipogenesis. Fact that over expression of E6AP in 3T3-L1 cells dramatically destabilizes endogenous C/EBPα protein and inhibits its transactivation capacity provides an explanation for inhibition of adipogenesis by E6AP. Note that, 3T3L1 cells are unique in a way that serum alone is not able to direct re entry of contact inhibited cells in cell cycle [32]. Several adipogenic agents (methylisobutylxanthine, dexamethasone and insulin) are required for induction of cell cycle in density dependent growth inhibited cells and thereby leading it to adipocytic differentiation [33]. Overexpression of catalytic domain mutant E6AP-C843A in mouse mesenchymal stem cells induces intracellular lipid droplet formation even in the absence of adipogenic factors which strongly indicates a role for E6AP in adipogenic differentiation. This is very interesting as mesenchymal progenitor cells are the pluripotent cells which can differentiate in varied lineages depending upon kind of hormonal inductions [34]. This apparently provides a clue that E6AP is an important regulatory switch in the adipocytic differentiation.

E6AP is encoded by *UBE3A* locus on chromosome 15. This chromosome 15 is important in various genetic disorders like Angelman syndrome (AS) [35] and Prader-Willi syndrome (PWS) [36]. In most of the Angelman syndrome cases there is deletion in maternal copy of 15q11-13 leading to mutation in UBE3A gene. Although, in PWS the exact role of UBE3A is not known but there is deletion in paternal copy of chromosome 15. However, one interesting phenotype reported both in AS and PWS patients is obesity. AS patients are more susceptible to gain weight [37], [38], while obesity is one of the major problems in PWS patients leading to higher mortality. The exact genes involved in these phenotypes are not yet known, and in fact there may be some genes located on chromosome 15 unreported thus far which may control obesity. Nonetheless, our data showing negative regulation of adipogenesis by E6AP via degradation of C/EBPα may also provide an explanation for increased body weight and obesity in these patients. However, this further needs to be investigated in more detail in those patients.

**Figure 6:**
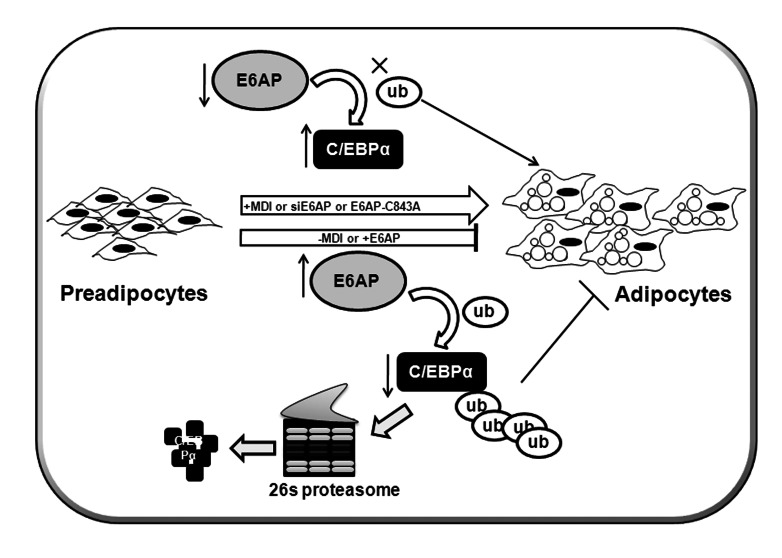
Hypothetical model for E6AP mediated negative regulation of adipogenesis. Depicts a hypothetical model suggesting higher expression of the E3 ubiquitin ligase E6AP in preadipocytes inhibits proadipogenic transcription factor C/EBPα apparently by targeting it for ubiquitin mediated degradation and thereby attenuating its transactivation potential and adipocyte differentiation.

Based on our data presented in this study we propose a hypothetical model (fig. 6) where we show that E6AP negatively regulates adipogenesis. High expression of the E3 ubiquitin ligase E6AP in preadipocytes may promote degradation of proadipogenic transcription factor C/EBPα, thereby attenuating its transactivation potential and thus adipocyte differentiation. Expression of E6AP is down-regulated early during adipogenesis, leading to the stabilization of C/EBPα, which in turn binds to the promoters of target genes and effectively results in increased expression of proadipogenic genes and enhanced adipogenesis.

Furthermore, since E6AP inhibits adipogenesis by downregulating C/EBPα via targeting it for ubiquitination and subsequent proteasome mediated degradation; its potential as a target protein in the treatment of obesity and type-2 diabetes may further be explored. Also, it is very important to identify the signals and factors involved in the adipogenesis which may regulate E6AP expression. In addition, since C/EBPα plays crucial role in many other cellular processes, role of E6AP in regulating C/EBPα functions in those processes may also be investigated.
